# Study protocol for a stepped-wedge, randomized controlled trial to evaluate implementation of a suicide risk identification model among behavioral health patients in three large health systems

**DOI:** 10.1186/s12888-025-06760-0

**Published:** 2025-04-08

**Authors:** Scott P. Stumbo, Stephanie A. Hooker, Rebecca C. Rossom, Kathleen Miley, Brian K. Ahmedani, Elizabeth Lockhart, Hsueh-Han Yeh, Bobbi Jo H. Yarborough

**Affiliations:** 1 Kaiser Permanente Center for Health Research, Portland, USA; 2 HealthPartners, Minneapolis, USA; 3 Henry Ford Health System, Detroit, USA

**Keywords:** Suicide risk identification, Suicide prevention, Implementation

## Abstract

**Background:**

Age-adjusted suicide rates have increased in the U.S. over the past 25 years. Algorithm-based methods for identifying individuals at risk for suicide based on electronic health record and claims data have been validated but few studies have evaluated implementation or effects on population-level suicide attempt rates.

**Methods:**

This hybrid type I effectiveness-implementation pragmatic clinical trial will test a suicide risk identification model in behavioral health clinics at three large health systems. Local decision-makers will determine implementation specifics at each site. Clinics within each health system will be randomized to determine order of implementation. A stepped-wedge design using repeated measures pre/post-implementation maximizes statistical efficiency and power with fewer participants compared to a parallel design while allowing all clinics to participate. A pre-implementation period will serve as the baseline. The primary outcome will be the rate of suicide attempt per 1000 visits at 90- and 180-days following a behavioral health visit in which an individual was identified by the suicide risk model compared with the baseline period (no use of suicide risk model). Secondary outcomes include identification of suicide risk and recognition of individuals at risk for suicide (e.g., completed risk assessment), both compared to the baseline period. Generalized linear mixed models will be used to account for clustering within clinics and repeated measures over time, adjusting for relevant covariates to estimate the effect of the suicide risk model on outcomes. Implementation outcomes, including system-level determinants and clinician acceptance and use of the suicide risk model, will also be measured.

**Conclusions:**

Few suicide risk models derived from administrative and clinical data have been tested in real world care settings. This trial will determine whether the use of such a risk model reduces suicide attempts compared to usual care. By describing important implementation factors, use of such risk models, if effective, may be accelerated for other health care systems.

**Trial registration:**

ClinicalTrials.gov NCT06060535.

**Supplementary Information:**

The online version contains supplementary material available at 10.1186/s12888-025-06760-0.

## Background

Despite local, state, and national prevention initiatives, age-adjusted suicide rates in the US increased by 37% from 1999 to 2018, then declined 5% from 2018 to 2020 but returned to near peak levels in 2021 and 2022 [1, 2]. Suicide remains one of the top ten leading causes of death among individuals aged 10–64 years old [3]. In 2022, more than 49,000 individuals in the U.S. died by suicide and 1.6 million adults attempted suicide [2]. Concern about rising suicide rates prompted the National Action Alliance for Suicide Prevention and the US Surgeon General to publish the 2024 *National Strategy for Suicide Prevention (NSSP)* [4]. According to the NSSP report, one of the most promising environments to implement suicide prevention practices is health care, where clinicians can be trained to detect suicide risk and intervene with effective care [5]. This is important given that, in one large study across eight health systems, > 83% of individuals who die by suicide and > 92% of those who attempt suicide had a health care visit within 12 months of the suicide event [6–8].

Over the last two decades numerous prevention approaches have been developed and implemented in health care settings, including a national movement to adopt strategies consistent with the Zero Suicide (ZS) framework [10–13]. While these tools have demonstrated ability to identify some individuals at risk, there are many barriers that may prevent identification of others. To fill these gaps and overcome barriers, the Mental Health Research Network (MHRN) [14] developed suicide risk identification models derived from electronic health records (EHR) and insurance claims data. Previous suicide risk models used samples consisting of military or veteran populations that were predominantly male [15, 16]. In contrast, the MHRN suicide risk identification models include data from approximately 20 million visits made by three million patients at seven large integrated health care systems, with broad diversity in sex, age, race, ethnicity, income, education and insurance [17]. The resulting models outperformed risk stratification based solely on the PHQ9 suicide ideation/self-harm question (item 9) and most other published EHR data-based models [16, 18, 19, 20–26] with C-statistics for the MHRN models ranging from 0.83 to 0.86 [17].

The MHRN risk model development and validation studies demonstrated accuracy but did not evaluate whether the models increase identification (screening) and recognition (risk assessment) or result in patients receiving more suicide prevention services (treatment) and fewer crisis services or making fewer suicide attempts. The current study is designed to answer these important implementation questions and to understand factors that could support rapid scale-up and widespread use should the risk model prove effective. This study combines the MHRN suicide risk identification model (to flag patients estimated to be at risk for suicide) and the ZS framework (to identify, engage, and treat patients identified at risk for suicide) in an innovative approach across three integrated health care systems’ behavioral health clinics to increase the reach and effectiveness of suicide prevention care models.

## Methods

### Objectives and hypotheses

The current study will test the implementation of the MHRN suicide risk model in behavioral health clinics to facilitate identification, recognition, and engagement in supportive suicide prevention services among individuals estimated to be at risk for suicide. The study’s main aim is to examine the effectiveness of a suicide risk model on fatal and non-fatal suicide attempts (primary outcome). The secondary outcome analyses examine the following questions: Are patients who receive care in the behavioral health intervention clinics more likely to be referred to and receive supportive outpatient behavioral health services than patients in usual care clinics? Are they more likely to receive supportive services sooner after risk recognition than they would have before the risk models were used, or earlier than patients in usual care clinics? Are they less likely to require crisis services and less likely to attempt suicide? Specifically, we will test the hypothesis that the risk model will lead to a reduction in suicide attempts (primary outcome) and an increase in referrals to, and receipt of, supportive outpatient services (secondary outcome).

In secondary analyses, we will determine whether implementing a suicide risk model into a suicide prevention care model improves identification of patients at risk for suicide attempt (suicide screening practices); that is, whether the enhanced identification using a risk model improves upon self-reported risk screening relied upon in usual care. Specifically, does the risk model (intervention) identify additional patients for screening who would not be identified in the course of usual care? How many additional patients are recognized (complete a risk assessment)? We hypothesize that the risk model may identify people who miss or refuse screening, deny risk because they do not believe themselves to be at risk or feel ambivalent, or deliberately conceal their risk. We will test the hypothesis that the suicide risk model implementation will improve identification and recognition of patients at increased risk of suicide attempt compared to usual care.

The study will also collect implementation data as part of the process of evaluating the intervention in three health systems. We will collect information during the start-up period (to document current usual care for suicide prevention and local implementation decisions), during the intervention period (to collect information on adaptations, should they occur), and during the sustainment period (to capture lessons learned and whether and how the intervention endures). Data will be collected in the form of meeting minutes, observations, and field reports from clinical leads and champions. Additionally, surveys of clinicians who will work with the suicide risk model intervention will be conducted prior to implementation and six months following implementation.

### Study settings

The study will be implemented in behavioral health clinics in three health care systems with a long history of collaborative work; all three systems were part of the original MHRN work to develop and validate the suicide risk models [17].

### Kaiser Permanente Northwest (KPNW)

KPNW includes 10 behavioral health clinics serving approximately 50,000 patients annually, a behavioral health inpatient hospital, and a dedicated ZS implementation team. In 2007, KPNW began screening all patients attending outpatient behavioral health office visits using the PHQ9. Strategies expanded to include the C-SSRS in 2012, an EHR version of the C-SSRS in 2016 and a revision in 2018 making the data more accessible for research and quality improvement. Safety planning, means counseling, caring contacts, and cognitive behavioral therapy (CBT) are standard care.

### Henry Ford Health (HFH)

HFH has four outpatient behavioral health clinics serving > 60,000 patients annually and four behavioral health inpatient hospitals. The department has a suicide prevention task force, which oversees implementation, evaluation, and quality improvement for the ZS Program, which have been implemented since 2001. Indeed, ZS was developed from the Perfect Depression Care [27–29] initiative at Henry Ford; this site has a long history of international leadership in suicide prevention. In 2018, HFH updated its ZS protocols, including use of universal screening along with suicide risk assessment, safety planning, caring contacts, care coordination, and a comprehensive means safety protocol. All psychotherapists are trained in CBT. The health system also implemented a dialectical behavioral therapy program for suicide prevention.

### HealthPartners (HP)

HP is an integrated health care system with 39 behavioral health clinics serving approximately 58,000 patients annually. HP has used the PHQ9 since 2007. More recently, HP behavioral health and primary care leadership has been active in a statewide collaborative to implement ZS led by the Institute for Clinical Systems Improvement. This has included a roll-out of a depression express lane in HP’s EHR, including integration of the C-SSRS and safety planning. The MHRN suicide risk model was incorporated into HP’s health plan’s care management workflow in 2019 and into a pilot study of a clinical decision support tool for patients with opioid use disorder in eight primary care clinics in 2021 [30], but it has not previously been more widely implemented into behavioral health clinical care.

The three sites provide important variability in size, organizational structure, and degree of ZS implementation that will enrich outcome and covariate comparisons in the study.

### Overview of study design

The current study will use a hybrid type I effectiveness-implementation approach to test implementation of an EHR-derived suicide risk model to facilitate identification, recognition, and engagement in supportive suicide prevention services among individuals who are estimated to be at risk for suicide and receiving services in behavioral health clinics at the participating health systems. This includes a stepped wedge cluster randomized trial design [31, 32] with randomization of behavioral health clinics into three waves across the three participating health systems. This design offers a novel approach to studying service delivery interventions as it provides a baseline period without implementation followed by random and sequential crossover of clinics from usual care control to intervention until all participating clinics are exposed. All clinics will have a pre-implementation phase (baseline period). Within that, just prior to implementation, health systems will train clinicians. Clinician training materials will be provided but sites will operationalize the training according to local customs and resources. Differences in training rigor provide additional variability as they may partially account for acceptability or feasibility. Implementation begins when each randomized clinic (stratified by site) transitions from usual care to running the risk model so clinicians can observe the risk indicator (the intervention is the enhanced care model with the suicide risk model and associated clinician notification). Analyses will examine within and between clinic variation in outcomes and account for time since implementation. This design allows implementation of the intervention at all clinics during the study period, a requirement of our clinical stakeholders; similar designs have been used in other suicide prevention trials [33, 34].

Given the novelty of this intervention, it is premature to propose testing implementation strategies as it is unclear which combination of strategies would best address local determinants and support implementation and effectiveness. Instead, we will leverage findings from our preliminary studies [35–38] in an implementation tool kit to help our clinical partners customize implementation plans for their local context. We will describe and measure use of the tool kit and implementation determinants, strategies, and outcomes hypothesized to impact the effectiveness of the intervention to prepare for future implementation work if the model proves successful. The tool kit will be revised based on adopter and clinician feedback.

### Clinic randomization and eligibility

**Fig. 1:**
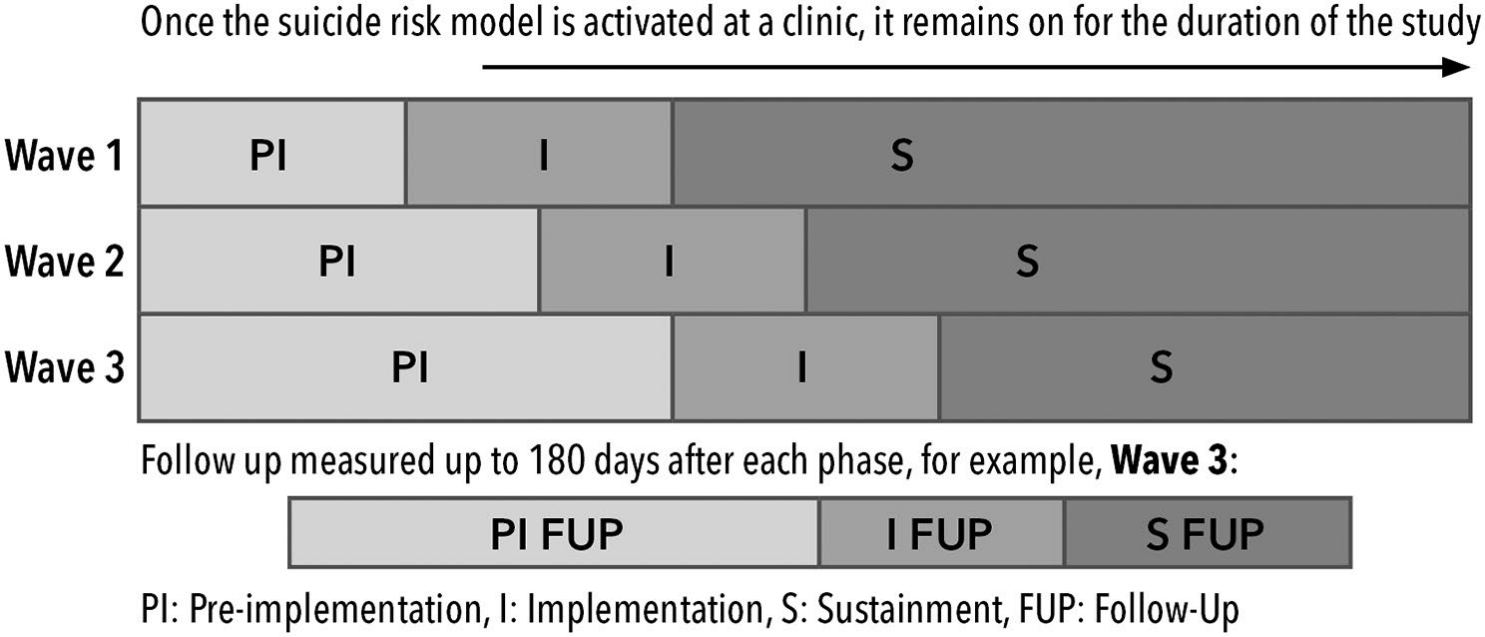
Stepped wedge design and study timeline

Sites will provide a list of behavioral health clinics to the lead analytical site (HFH). The goal is to randomize clinics into two or three waves each starting approximately three to six months apart with all waves eventually participating in the intervention (see Fig. 1). Randomization across sites will be conducted in SAS 9.4 with a simple randomization procedure (PROC SURVEYSELECT), to determine which clinics will enter the implementation period first.

All individuals being seen in behavioral health care in one of the participating clinics are eligible for the trial, there are no exclusion criteria. Individuals included in the trial are not prohibited from seeking any care or services. Enrollment is anticipated to end July 2025.

### Implementation conceptual framework

**Fig. 2:**
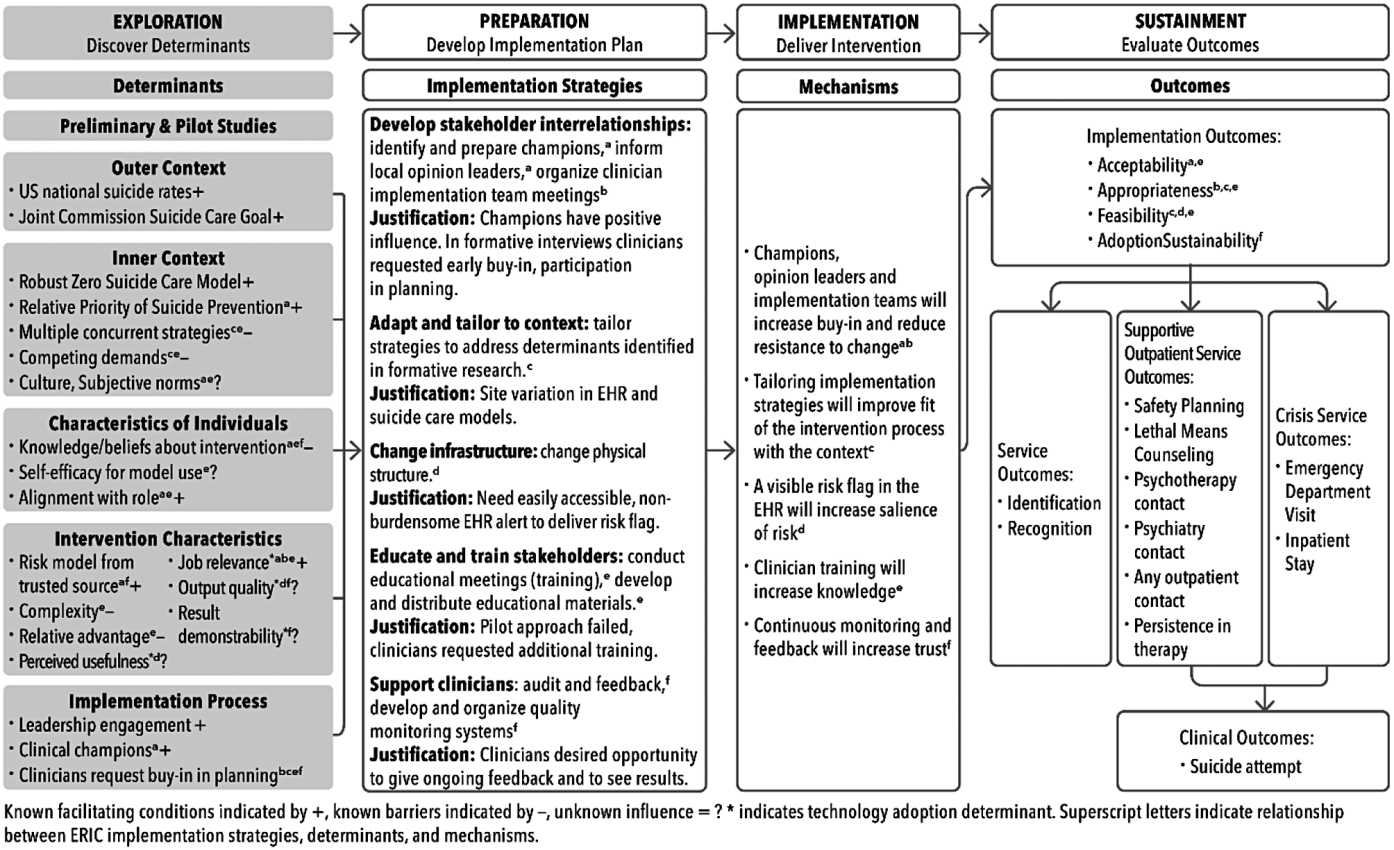
Implementation research logic model

We will use the Exploration, Preparation, Implementation, and Sustainment (EPIS) framework [39] to guide implementation planning, execution, and evaluation. This framework acknowledges that context influences implementation differently during different implementation phases and over time. This framework will help us focus on what is important as we move from planning to implementation to the process evaluation. Prior to the current study, we began the *Exploration* phase with our preliminary work [37, 38] focused on understanding determinants (within CFIR [40] categories) likely to influence suicide risk model implementation. *Preparation* will continue in year 1 among the implementation teams. Many preparations including ascertaining leadership support and identifying clinical champions, familiarizing leadership with preliminary findings related to patient and clinician preferences and recommendations for implementation, and anticipating technological and logistical requirements to enable the risk model in the EHR were already in process at the study sites prior to the study’s start. In year 1, each site will be encouraged to develop an Implementation Research Logic Model (IRLM) [41] and detailed implementation plan (see example in Fig. 2, with EPIS stages overlaid) with identified clinic champions. The IRLMs will specify known implementation determinants at each site, strategies to address the determinants, and the implementation, service, and clinical outcomes. For completeness, Fig. 2 (for illustration purposes only) also includes putative mechanisms through which the strategies may achieve the effectiveness and implementation outcomes.

### Proposed care models (mechanisms of action)

**Fig. 3:**
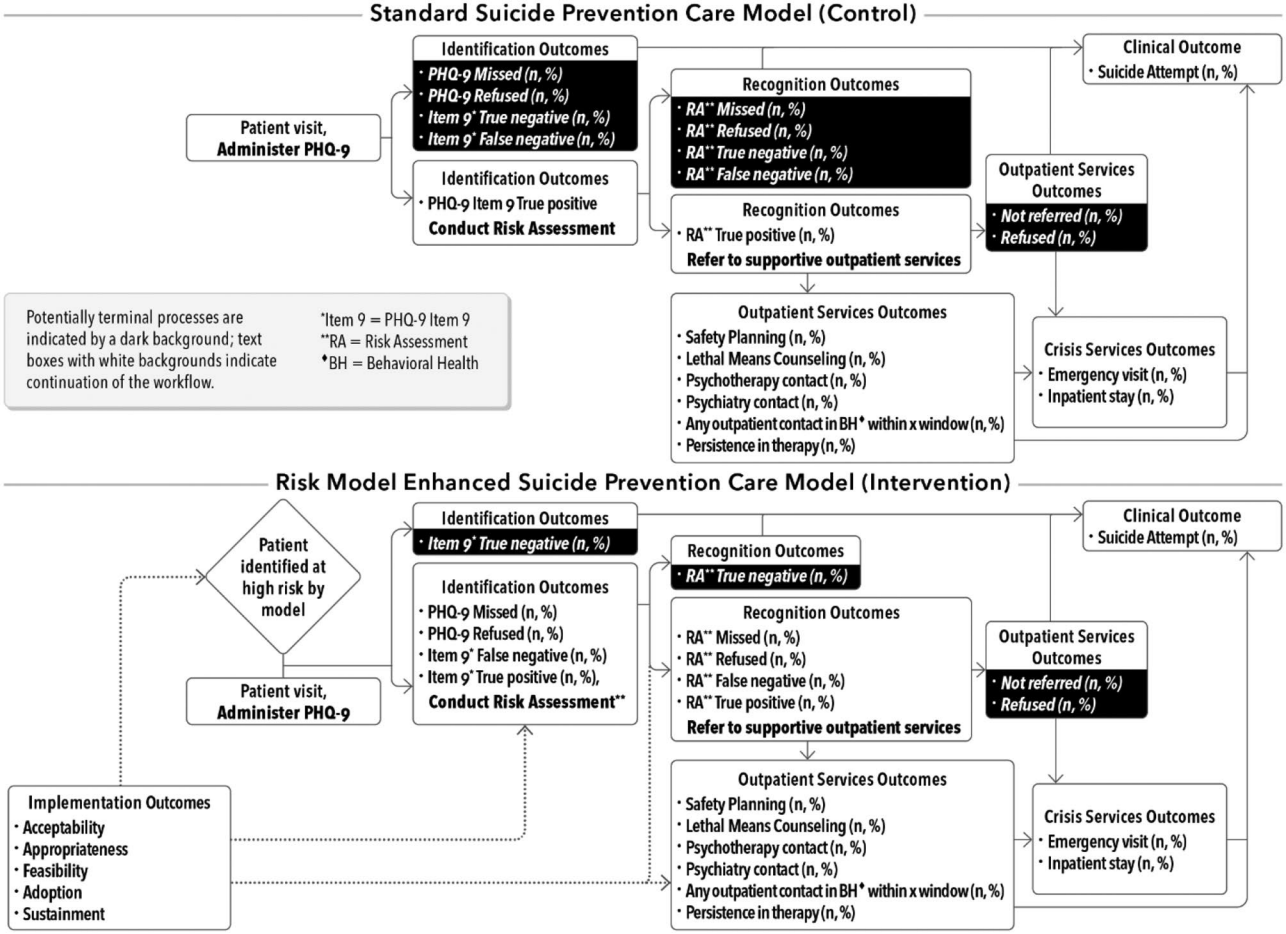
Comparison of care models

Figure 3 shows a standard suicide prevention care model (usual care) and the proposed enhanced care suicide risk model (intervention). A typical workflow that involves suicide risk identification, when a patient presents for a behavioral health visit, may begin with suicide risk screening (typically the PHQ9 [42] in the participating sites). If the PHQ9 is missed, refused, or if item 9 (“Over the last two weeks, how often have you been bothered by thoughts that you would be better off dead or of hurting yourself in some way?”) is denied, the workflow may terminate, however a clinician could also proceed if clinically indicated. Potentially terminal processes are indicated by a dark background in Fig. 3, text boxes with white backgrounds indicate continuation of the workflow. The expectation is that when patients respond positively to item 9 (i.e., respond with a score of 2 “more than half the days” or 3 “nearly every day”), a comprehensive suicide risk assessment is conducted (typically the Columbia Suicide Severity Rating Scale [C-SSRS] [43] in the participating sites). Again, if the risk assessment is missed, refused, or if risk is denied the workflow may terminate. The expectation is that when risk is confirmed, depending on acuity, a referral is made to outpatient supportive services or to the emergency department or inpatient setting.

The enhanced care suicide risk model (intervention) demonstrates the proposed changes in workflow with the risk model implementation. In this model, the suicide risk model flags the clinician in the EHR to conduct additional suicide risk screening. The expectation is that if the PHQ9 were missed, refused, or if item 9 was denied in the presence of a model-generated risk flag indicating elevated estimated risk, then the clinician would proceed to the C-SSRS as if the screening were positive.

It is important to note that among patients who present for behavioral health services, some will have minimal or no suicide risk, some will be thought to be at *clinical* risk (i.e., the treating clinician estimates the patient is at risk) [44] but will not be flagged at *estimated statistical* risk by the risk model, some will be flagged at estimated statistical risk but will deny acute clinical risk (e.g., some may have enduring suicide ideation that is being treated), and some flagged at estimated statistical risk will be confirmed, upon assessment, to be at acute clinical risk (for some this will be an incident recognition, others will already be identified and perhaps treated). The goal of the model is to prompt risk assessment of anyone determined to be at estimated elevated statistical risk, and to prompt supportive preventive services when appropriate. How care proceeds after the risk assessment is dependent on the outcome of the risk assessment and the clinician’s judgement. This is an important detail because clinicians and patients have expressed concern about risk models having the potential to lead to coercive or inappropriate treatment; the risk model prompts identification and recognition of risk but is agnostic to treatment decisions thereafter.

### Data sources

Primary and secondary outcomes will be derived from EHR and administrative claims data. As members of the MHRN, all participating health systems have complete capture of EHR and claims data for their patients [14]. Additionally, all MHRN sites have structured their electronic health records and insurance claims data into locally maintained federated data systems known as the Virtual Data Warehouse (VDW) [45] at each site to support comprehensive and efficient capture of health care encounters, diagnoses, medications, patient-reported outcomes, demographics and other administrative and clinical data. We will leverage these data sources and previously validated MHRN definitions [46] for measurement of our variables. Implementation outcomes will be derived from meeting minutes, observations, field reports, and survey data collected from clinicians in participating clinics.

### Study measures and effectiveness outcomes

The primary outcome measure is fatal or non-fatal suicide attempt (binary yes/no) within 90 or 180 days of a behavioral health visit as measured using diagnosis codes for intentional self-harm (International Statistical Classification of Diseases and Related Health Problems, Tenth Revision [ICD-10] codes X60-X84; Y87.0). Suicide deaths will be ascertained from government mortality records. The denominator will be the total number of visits to behavioral health clinics and the numerator will be reported as the number of suicide attempts per 1000 visits. In addition to the primary outcome, we will examine proximal outcomes and crisis utilization measures. Proximal outcomes include measures of engagement with services documented in the EHR within the observation period. These are defined as: evidence-based suicide prevention interventions (safety planning, lethal means counseling, or caring contacts) on or after the risk identification (index) encounter, an outpatient encounter (includes telephone, virtual, and office visits) by a behavioral health clinician or psychiatrist subsequent to the index encounter, any contact with behavioral health within seven or 14 days of the index visit (includes phone calls, letters, patient portal messages, or other caring contacts in addition to encounters described above), frequency of contacts, latency to next encounter after index, and persistence in psychotherapy defined as no gap in psychotherapy visits > 90 days during the observation period. Crisis utilization measures include any encounters in the emergency department or inpatient stays subsequent to the index encounter within 90 days and 180 days.

Secondary outcomes include suicide risk identification and recognition. Identification will be measured by the number and proportion of patients estimated to be at elevated risk by PHQ9 item 9, the risk model, or both, stratified by race/ethnicity. This will be assessed at each visit, as multiple visits may occur for the same individual. However, when calculating identification and recognition, we will aggregate visits at the individual level—categorizing outcomes as yes/no for binary measures and recording the number of identifications and recognitions as count outcomes. Aggregated assessment results will be generated at 90 and 180 days. Therefore, the denominator is the number of unique patients with an encounter in the study period. In the intervention clinics we will also measure the number of patients flagged by the risk model who, at the index encounter, did not complete the PHQ9 or denied item 9. Those identified by the risk flag but not through traditional PHQ-9 will represent the gain from using a model-based identification approach; in the usual care model these patients would not be identified as having elevated estimated increased risk. Suicide risk recognition will be measured by the number and proportion of patients who complete a risk assessment (C-SSRS), stratified by race/ethnicity, where the denominator is the number of unique patients with an encounter in the study period. We will also measure the proportion who completed a C-SSRS but did not have an elevated PHQ9 item 9 score but were estimated to be at elevated risk by the suicide risk model (for comparison, this will also be measured among usual care control participants as some patients will receive risk assessments independent of prompting by PHQ9 item 9 score).

### Sample size and power analysis

We anticipate including > 50 clinics and approximately 395,000 unique behavioral health patients across three sites over a 2.5-year period, leading to over 1 million patient visits. We project that approximately 4–5% of patients will be identified as high risk and thus eligible for the intervention. Utilizing a stepped-wedge cluster randomized controlled trial design, with > 50 clinics randomly assigned across three waves, we will assess the primary outcome, suicide attempts, at 90- and 180-days post-intervention. Given an anticipated suicide attempt rate of 0.6% (*n* = 2,370) within 90 days of a visit, a 0.01 intraclass correlation coefficient within waves, we expect to achieve more than 80% power (2-sided *p* <.05) to detect an effect size corresponding to a 1.5% absolute difference in suicide attempt rates [47–49].

### Analysis plan for effectiveness outcomes

We will analyze outcomes, for both: (1) unique patients, defined as those new to the study sample without a visit in the past three months, and (2) unique visits, where each health care encounter is considered separately, allowing for multiple visits per unique person, given that patients may be screened or identified as at-risk at multiple visits.

Only study biostatisticians will have access to the final trial datasets. We will begin with descriptive analyses of the study sample, including patient demographic characteristics, encounters, history of past-year suicide attempt, and current mental health diagnoses by exposure group. Descriptive analyses will also examine each of the individual identification and recognition measures using health system records, stratified by exposed (intervention) versus unexposed (control) periods. We will calculate the clinic/cluster sizes for each phase of implementation and clinic/cluster characteristics by exposure period to assess balance across the study period. Standard chi-square tests and t-tests will be used to compare variation in each outcome variable between exposure groups, and in each unique phase of implementation. We will then plot change in both counts and proportions of each individual outcome graphically by study exposure period.

Generalized Linear Mixed Models (GLMMs) with logit link function will be used to assess categorical outcomes (identification and recognition, separately, defined as yes/no) to study the effect of the intervention on identification and recognition. The results will be presented as odds ratios (OR) with 95% CIs. We will analyze the frequency of events identified through PHQ-9 (identification), recognized through the C-SSRS (recognition), or both combined. Additionally, GLMMs with a log link function will be used to separately assess the effects on identification and recognition by modeling the count of identified or recognized patients. The model will include the intervention (yes/no) as an independent variable to assess its effect while accounting for clustering and potential confounders. Among visits where individuals are identified or recognized as being at risk, we will further investigate factors influencing these outcomes, including patient demographics and clinical characteristics. The results will be presented as relative risk (RR) with 95% CIs. In these analyses, clusters will be analyzed according to their randomized crossover time. Models will include a random effect for cluster and a fixed effect for each phase, and will adjust for age, sex, race/ethnicity and cluster/clinic terms (i.e., secular trend). These models are optimal and commonly used for studies implementing stepped wedge cluster randomized trial designs [31, 32, 50]. All analyses will also adjust for time, given that the ratio of exposed clinics increases over time, and calendar month to account for seasonality of suicide attempts. Given that patients may be screened or identified as at-risk multiple times, the frequency of visits during the follow-up period will be adjusted in the model as well. In additional analyses, the model will account for cluster effects by clinic and correlation between repeated measurements for the same patient over time.

These models will also be used to test mediators/mechanisms, given that we hypothesize that identification alone will not reduce suicide outcomes but rather increase the number of people receiving suicide intervention, which could lead to improvement (i.e., decrease) in suicide outcomes. Mechanisms include interaction with the alert and initiation of assessment, which starts the outpatient care pathway. We will also stratify analyses by health system site, and test for mental health diagnoses and prior suicide attempts as moderators. Importantly, we will exclude ‘training’ months for each phase of clinic implementation from the main analyses, since clinicians and patients may be partially exposed as the models are implemented and clinicians trained. In a secondary set of analyses, we will explore potential heterogeneity in treatment effects between clinics. Here we will conduct additional analyses to test within cluster comparisons of exposed and unexposed periods.

To examine the effectiveness of a suicide risk model on health service engagement and suicide attempt outcomes, we will start with similar descriptive and GLMMs analyses proposed previously for identification and recognition. The analyses here will focus on each unique health service engagement (supportive and crisis encounters separately) and suicide attempt outcomes, stratified by exposed (intervention) versus unexposed (control) groups. Suicide attempts will be captured by health system records using ICD-10 diagnosis codes occurring within the same 90-day and 180-day time periods. We will also use a Kaplan-Meier method and Cox-proportional hazards models to examine time-to-event for health service engagement and suicide attempts, with statistical adjustment for clinical and demographic factors. Individuals will be censored at the time of death or at 180 days past the index visit. We will again add terms for calendar month as well as site to account for variation by season and health system.

Finally, we will separately examine the impact of health care engagement and exposure to ZS evidence-based interventions as mediators of suicide attempt and use of crisis services. We will estimate direct and indirect effects in our longitudinal settings using our previously proposed GLMMs [51]. Specifically, we will test the effect of the intervention (risk model study group, yes/no) on behavioral health service engagement (yes/no within 7 and 14 days via path ‘*a’*) and the effect of behavioral health service engagement on suicide attempt outcome (yes/no within 90 and 180 days via path ‘*b*’). The residual effect of the intervention unaccounted for by behavioral health service engagement will be tested via path ‘*c*’. We will also test a separate path model of exposure to documented suicide prevention interventions and their impact on suicide attempt outcomes using the same modeling approach. These models will include both individual documented supportive outpatient interventions for suicide and multiple interventions bundled together; we will also repeat these analyses for crisis services outcomes.

We will address missing data using multiple imputation or inverse probability weighting, depending on the nature and extent of missingness. Additionally, if the proportion of missing data is substantial, we will conduct sensitivity analyses to assess its potential impact on our findings.

### Implementation outcomes

There is little published guidance on how suicide risk models should be implemented in clinical settings. Work we completed in preparation for this implementation trial gave us insights into what patients and clinicians thought about the possible use of such models in clinical care [35–38]. The Technology Acceptance Model [52] posits that perceived usefulness of a technology positively influences use intention, which then drives adoption [53]. Perceived usefulness is one aspect of appropriateness of an intervention, and together these operate among many interacting components (e.g., burden, ethicality and fit with value system, knowledge) to influence acceptability [54], a necessary but insufficient condition for use and effectiveness. Beliefs about appropriateness and acceptability are influenced by the sociocultural context and perceived norms within which the technology user operates [53, 54]. Of course, feasibility of an intervention also affects use. Use is a precondition for reach and effectiveness, and acceptability, feasibility, and effectiveness may contribute to overall sustainability.

We will measure these outcomes by surveying clinicians who will be exposed to the intervention (e.g., therapists, psychologists, psychiatrists) prospectively (2–4 weeks prior to implementation) and concurrently with use (six months after implementation begins). To reduce clinician burden, we chose a parsimonious set of validated implementation measures (< 40 items). The Perceived Usefulness Scale [55] consists of 6 items with high reliability [Cronbach’s α = 0.98] and high convergent, discriminant, and factorial validity. The Intervention Appropriateness Measure [56] (IAM, 4 items), Acceptability of Intervention Measure [56] (AIM, 4 items), and Feasibility of Intervention Measure [56] (FIM, 4 items) trio have demonstrated content validity, discriminant content validity, reliability, structural validity, structural invariance, known-groups validity, and responsiveness to change. Higher scores indicate greater acceptability, appropriateness, and feasibility. The Intervention Usability Scale [57] (IUS, adapted from the System Usability Scale [58], 10 items) can effectively differentiate between usable and unusable interventions. A score of > 70 will be considered adequate usability. And, pre-implementation only, the Organizational Readiness to Change [59] (ORIC) scale measures an organization’s readiness to implement a change to their current processes; higher scores indicate greater readiness. Adoption will be measured at the clinic level as the proportion of clinics (out of total clinics) that implement the risk model, with 75% as a benchmark. Sustainment will be measured six months after the intervention phase ends by determining the number of clinics continuing to use the risk model, with 75% as a benchmark.

### Analysis plan for implementation outcomes

We will use descriptive statistics (frequencies and crosstabs) to summarize and compare clinician survey results prior to and following six months of intervention use. To compare clinician sentiment about the use of suicide risk models in clinical care we will use General Linear Models for repeated measures using the Greenhouse-Geisser test for within-subjects effects. We will document pre-implementation decision-making and adaptions during the implementation period, should they occur, in a series of reports.

### Ethical considerations

The Northwest Permanente Prediction Model Governance Committee reviewed and approved implementation of the model in KPNW. The Kaiser Permanente Interregional Institutional Review Board (IRB) has reviewed and approved the study protocol and will review any subsequent amendments. All sites have ceded authority to the IRB for ongoing monitoring of the research aspects of the project. A waiver of informed consent was granted as the sample size makes consenting individuals impractical and only secondary data will be used for the effectiveness analyses. The waiver allows sites access to identifying information at the local level; however the data will be anonymized prior to the pooled analysis. Clinicians will be sent a study information sheet prior to recruitment for the implementation surveys; it will be made clear to them that by submitting a survey they are consenting to participate in the study. All EHR and claims data from the three participating sites will be stored locally. Only de-identified datasets will be shared between sites under appropriate data use, sharing, and transfer compliance agreements. All project staff who work on research projects and handle human subjects’ data or have human subjects contact, sign confidentiality agreements and receive IRB and HIPAA training/certification.

We will convene an independent Data and Safety Monitoring Board (DSMB) composed of a Chair and at least two members; the chair and at least one member will have extensive expertise in suicide prevention in health care settings, at least one member will have statistical expertise in stepped-wedge design and pragmatic trials. The DSMB will create a charter, maintained by the Chair, and will meet twice annually during the study to discuss recruitment progress and any reported safety concerns. Interim analyses for the DSMB meeting will include patient characteristics by implementation phase and wave for all sites and a quarterly count of individuals with a suicide attempt within 90 days of a visit and 180 days of follow up across all sites. The latter allows for surveillance of safety by looking at overall patterns of suicide attempts without examining the main outcome prior to the formal study analyses. The DSMB will have access to interim results to make decisions regarding premature termination of the trial. The site project managers will also solicit from clinic champions and report adverse events or unintended consequences of the trial or trial conduct, and these will be presented to the DSMB.

### Creation of an implementation tool kit

The process evaluation for the study will culminate in an implementation tool kit for health care leaders considering adoption of suicide risk models. The tool kit will include summaries of the findings from the preliminary [35, 37, 38] and current studies; the ethical framework to guide implementation decision making [36]; guidance for consideration in selecting risk thresholds for action based on clinical learnings that emerge during implementation; detailed contextual descriptions of implementing sites (e.g., setting, resources, existing suicide prevention care model and infrastructure supports) as case examples; descriptions of local determinants (e.g., barriers and facilitators) and whether and how much they were modified by the implementation strategies over time; descriptions of implementation strategies and plans that were considered and executed, including adaptations and reasons for adaptations; the final IRLMs for each site; training materials and resources; and estimated staffing allocations necessary for key roles (e.g., clinic champions, programmers, etc.).

### Dissemination of study results

Early and ongoing implementation findings and eventually trial results will be communicated to the participating sites through the clinical champions and implementation teams and will be included in a revised tool kit. Study results will further be communicated through manuscripts in peer-reviewed journals, national conferences, and through several networks that study investigators routinely collaborate with (e.g., Mental Health Research Network, Kaiser Permanente National Suicide Learning Collaborative, Suicide Prevention Resource Center, Zero Suicide International). Consistent with our values of collaboration and transparency, we will make our documentation, research methods, protocol, publicly available data collection tools and, upon completion of analyses and publications, a de-identified dataset (redacted to prevent re-identification) available upon request to interested researchers for non-commercial research use.

### Potential study limitations

This study will be conducted in integrated health care systems and findings may not generalize to other settings. Assessment of suicide attempts using EHR and claims data will reduce bias associated with patient report but may miss suicide attempts for patients who do not seek care following a suicide attempt if the attempt is not later recorded in the EHR or their attempt is not discoverable in the EHR. The study will identify at-risk patients who present for behavioral health visits but will not identify patients who are not engaged in these services nor patients who only present for care in other settings, such as primary care clinics or behavioral health clinics external to the three participating health care systems. Finally, the proposed stepped wedge study design is not as methodologically rigorous as a true cluster-randomized design, but this design was chosen in partnership with health system leaders, who did not want to delay implementation across behavioral health clinics longer than necessary.

## Conclusion

Few suicide risk models derived from administrative and clinical data have been tested in real world care settings. This trial will determine whether the use of such a risk model reduces suicide attempts compared to usual care. By describing important implementation factors, use of such risk models, if effective, may be accelerated for other health care systems.

## Electronic supplementary material

Below is the link to the electronic supplementary material.

Supplementary Material 1

## Data Availability

No datasets were generated or analysed during the current study.

## Abbreviations

C-SSRS: Columbia-Suicide Severity Rating Scale
CBT: Cognitive Behavioral Therapy
CFIR: Consolidated Framework for Implementation Research
DSMB: Data and Safety Monitoring Board
HER: Electronic Health Record
EPIS: Exploration, Preparation, Implementation, and Sustainment
GLMMs: Generalized Linear Mixed Models
HFH: Henry Ford Health
HIPAA: Health Insurance Portability and Accountability Act
HP: HealthPartners
ICD-10: International Statistical Classification of Diseases and Related Health Problems, Tenth Revision
IRB: Institutional Review Board
IRLM: Implementation Research Logic Model
MHRN: Mental Health Research Network
NSSP: National Strategy for Suicide Prevention
OR: Odds Ratio
ORIC: Organizational Readiness to Change
PHQ9: Patient Health Questionnaire-9
RR: Relative Risk
SUS: System Usability Scale
US: United States
VDW: Virtual Data Warehouse
ZS: Zero Suicide
